# Clinical factors associated with prognosis in low-grade serous ovarian carcinoma: experiences at two large academic institutions in Korea and Taiwan

**DOI:** 10.1038/s41598-020-77075-1

**Published:** 2020-11-17

**Authors:** Jun-Hyeok Kang, Yen-Ling Lai, Wen-Fang Cheng, Hyun-Soo Kim, Kuan-Ting Kuo, Yu-Li Chen, Yoo-Young Lee

**Affiliations:** 1 Division of Gynecologic Oncology, Department of Obstetrics and Gynecology, Samsung Medical Center, Sungkyunkwan University School of Medicine, 81, Irwon-ro, Gangnam-gu, Seoul, Republic of Korea; 2 Department of Obstetrics and Gynecology, National Taiwan University Hospital, Hsin-Chu Branch, Hsin-Chu, Taiwan; 3 Department of Obstetrics and Gynecology, College of Medicine, National Taiwan University, Taipei, 100 Taiwan; 4 Department of Pathology and Translation Genomics, Samsung Medical Center, Sungkyunkwan University School of Medicine, Seoul, Republic of Korea; 5 Department of Pathology and Graduate Institute of Pathology, College of Medicine, National Taiwan University, Taipei, Taiwan; 6 Department of Pathology and Laboratory Medicine, National Taiwan University Hospital, Hsin-Chu Biomedical Park Branch, Hsin-Chu, Taiwan

**Keywords:** Oncology, Risk factors

## Abstract

Low-grade ovarian serous carcinoma (LGSOC) has clinical features different from high-grade serous ovarian carcinoma (HGSOC) accounting for the majority of epithelial ovarian cancer. Because of its rarity, previous studies have only focused on the high-grade disease without considering the differences between the two subtypes. This study aimed to evaluate the effect of the clinical prognostic factors known for HGSOC on survival in patients with LGSOC. Based on the Federation of Gynecology and Obstetrics (FIGO) stage, progression-free survival (PFS) was markedly decreased in advanced disease compared with early disease. For stage I, patients with stage IC had poorer survival than those with stage IA and IB regardless of the number of cycles of adjuvant chemotherapy. For advanced disease, no gross residual disease after primary cytoreductive surgery was significantly associated with longer PFS when compared with gross residual disease. In multivariate analysis for PFS and overall survival (OS), age, preoperative CA-125, time interval from surgery to chemotherapy, and the number of cycles of adjuvant chemotherapy were not associated with prognosis. Complete cytoreduction was the only independent prognostic factor for PFS (HR 2.45, *p* = 0.045). Our study revealed that the known prognostic factors in HGSOC did not show any effect on the survival in LGSOC except for FIGO stage and complete cytoreduction.

## Introduction

Epithelial ovarian cancer (EOC) is the most lethal gynecologic cancer, as the majority of patients (75%) are diagnosed at an advanced stage owing to the lack of effective screening methods and vague symptoms^1,2^. EOC is composed of various histologic subtypes including serous, mucinous, endometrioid, and clear cell. Of these, serous ovarian cancer, the most common histologic subtype (75–80%), is divided into high-grade serous ovarian carcinoma (HGSOC) and low-grade serous ovarian carcinoma (LGSOC) according to nuclear atypia and mitosis rate in a two-tier system^3,4^.

LGSOC is a relatively rare cancer, accounting for only 5–10% of all EOC^5^. LGSOC has several clinical features that differ from the high-grade disease in terms of age at diagnosis, growing pattern of the tumor, and response to chemotherapy^5–8^. Patients with low-grade disease also usually show more favorable prognosis despite having similar stage distribution and are mostly detected at an advanced stage (Federation of Gynecology and Obstetrics (FIGO) stage III and IV), similar to the high-grade disease^9^.

The gold standard treatment for EOC is surgical debulking with or without platinum-based adjuvant chemotherapy according to the FIGO stage ± targeted agents (e.g., PARPi based on FIGO stage and *BRCA* 1/2 mutations or bevacizumab for high risk disease). Various clinical factors associated with the prognosis have also been revealed in previous studies such as age, tumor marker, residual disease after surgery, and the time interval from surgery to chemotherapy (TTC)^10–14^. However, many previous studies have been performed only focused on the high-grade disease or without distinction between the two histologic subtypes even though they have different characteristics. There are questions on whether it would be appropriate to apply the same criteria to predict the prognosis of the low-grade disease. Nevertheless, many physicians still use the same treatment strategy and criteria as those of the high-grade disease when managing LGSOC, because there are only a few studies about this issue and no standard guidelines have been established.

The purpose of this study was to evaluate the effect of known clinical prognostic factors for HGSOC on survival in patients with LGSOC.

## Methods

We retrospectively reviewed patients who were histologically confirmed with low-grade (grade I) serous ovarian cancer between 2000 and 2018 at the Department of Obstetrics and Gynecology at Samsung Medical Center, Seoul, Korea, and National Taiwan University Hospital, Taipei, Taiwan. Variables included patient demographics, tumor characteristics, first-line adjuvant chemotherapy, the time interval between surgery and disease progression or recurrence, and the follow-up period and survival. This study was done in accordance with the ethical principles and approved by the Institutional Review Board (IRB) of Samsung Medical Center and National Taiwan University Hospital (Ethical approval code: SMC2020-02-048 and 201902038RIND). The informed consent was exempted under the approval of the ethics committee.

**Figure 1:**
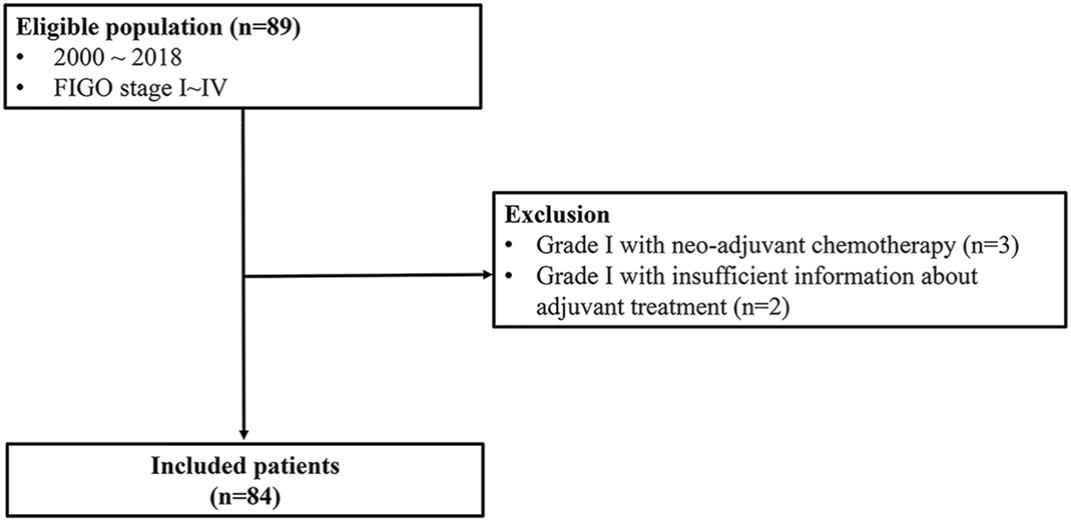
Inclusion and exclusion criteria for the studied population.

Patients were eligible for inclusion if they were diagnosed with LGSOC and FIGO stage I–IV, underwent primary surgery with or without platinum-based adjuvant chemotherapy, and had complete information regarding chemotherapy status and available follow-up data. LGSOC was defined as histologic grade I according to a two-tier system^3^. The exclusion criteria were as follows: grade 2 or grade 3 serous ovarian cancer, non-serous histology, received neo-adjuvant chemotherapy, and lack of information about the time interval between surgery and chemotherapy (Fig. 1).

Variables for age at diagnosis, year of diagnosis and preoperative CA-125 level were collected for patient’s baseline characteristics. Cancer stage was defined according to the 2014 International FIGO staging system. Residual disease after surgery was defined as no gross residual in case of no macroscopic residual disease, optimal, if the largest diameter of residual disease was < 1 cm, and suboptimal, if the largest diameter of residual disease ≥ 1 cm. The characteristics of chemotherapy included the chemotherapy regimen (platinum-based chemotherapy or not), number of adjuvant chemotherapy cycles (None, one to three cycles, and four or more), and the time interval from surgery to initial chemotherapy (time to chemotherapy, TTC). TTC was defined as the period between the date of the primary surgery and the initiation date of subsequent chemotherapy. PFS was defined as the date of the first treatment until progression, recurrence, death, or follow-up loss, whichever occurred first. OS was defined as the time interval from the day of surgery to the date of death or last contact.

Normality of the data was analyzed with the Shapiro–Wilk test. The mean ± standard deviation (SD) of the data was used for normal distributions, and the median (interquartile range, IQR) was used for non-normal distributions. Frequency distributions among categorical variables for the four stage groups were compared using the chi-square test or Fisher’s exact test. Quantitative variables were compared using one-way analysis of variance (ANOVA) as a parametric test or the Kruskal–Wallis test as a non-parametric test. Survival curves were calculated according to the Kaplan–Meier method with the log-rank test. The Cox proportional hazards model was used for multivariate analysis to assess the independence of different prognostic factors. *p* < 0.05 was considered to indicate statistical significance. Statistical analysis was performed using SPSS software (IBM SPSS Statics for Windows, version 25.0. Armonk, NY: IBM Corp. Available at: https://www.ibm.com/analytics/spss-statistics-software).

## Results

During the study period (2000–2018), a total of 84 patients were included in this study. FIGO stage III was most common (44/84, 52.4%) followed by stage I (30/84, 35.7%). Mean age of all patients was 49.0 ± 15.3 years (mean ± SD), and the median interval of TTC was 12 days (interquartile range 9–18).

**Figure 2:**
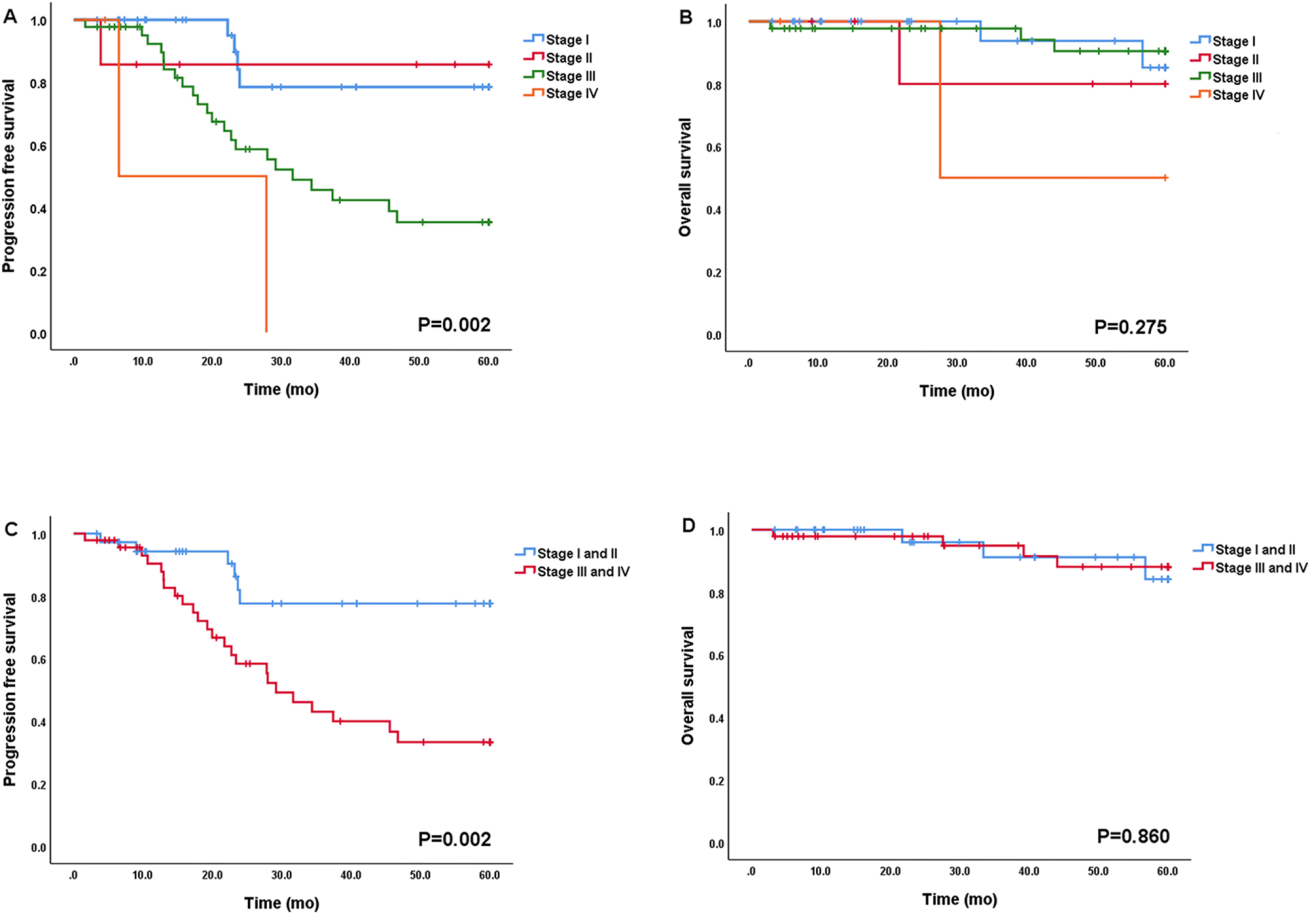
Progression-free survival and overall survival according to FIGO stage (all patients [n = 84]).

The patient and treatment characteristics according to the FIGO stage are summarized in Table 1. (Details of patients from each institution are shown in Supplementary Table S1). There was no significant difference in age according to the FIGO stage (*p* = 0.292). CA-125 levels were observed to be significantly higher as the FIGO stage advanced (*p* = 0.019). TTC was longer in advanced FIGO stage than in early FIGO stage, however, there was no significant difference (*p* = 0.293). Patients with advanced FIGO stage had more cycles of chemotherapy than patients with early FIGO stage (*p* = 0.001). As shown in Fig. 2, patients with advanced FIGO stage showed significantly shorter median PFS than patients with early disease (median PFS; stage I and II had not reached median PFS, stage III and IV, 20.2 and 6.5, *p* = 0.002; Fig. 2A,C). However, it was not statistically significant in OS (Fig. 2B,D). In addition, there was no difference in survival outcomes according to the difference in institutions (Supplementary Fig. S1).

**Table 1:** Patients and treatment characteristics.

Variable	Total (n = 84)	Stage I (n = 30)	Stage II (n = 7)	Stage III (n = 44)	Stage IV (n = 3)	*p*-value
**Age(years)**						
Mean ± SD	49.0 ± 15.3	46.5 ± 17.4	44.4 ± 15.2	50.6 ± 13.7	61.3 ± 19.8	0.292
**CA-125(U/mL)**						
Median(IQR)	298.7 (40.0–1368.9)	49.9 (12.1–151.8)	247.2 (84.4–315.1)	658.3 (150.8–1960.3)	2108.4 (2100.0–4590.3)	0.019*
**Residual disease**						0.002*
No gross residual	62 (73.8%)	30 (100%)	7 (100%)	24 (54.5%)	1 (33.3%)	
Optimal (< 1 cm)	12 (14.3%)	0	0	11 (25.0%)	1 (33.3%)	
Suboptimal (≥ 1 cm)	10 (11.9%)	0	0	9 (20.5%)	1 (33.3%)	
**TTC (days)**						
Median(IQR)	12 (9–18)	12 (10–18)	10 (9–17)	12 (9–17)	28 (21–34)	0.293
**Number of cycles of CTx**						0.001*
None	14 (16.7%)	11 (36.7%)	1 (14.3%)	2 (4.5%)	0	
1–3 cycles	12 (14.3%)	8 (26.6%)	0	3 (6.8%)	1 (33.3%)	
4 or more cycles	58 (69.0%)	11 (36.7%)	6 (85.7%)	39 (88.7%)	2 (66.7%)	

*TTC* time from the surgery to adjuvant chemotherapy, *IQR* interquartile range, *CTx* chemotherapy.

*p-value is less than 0.05.

**Figure 3:**
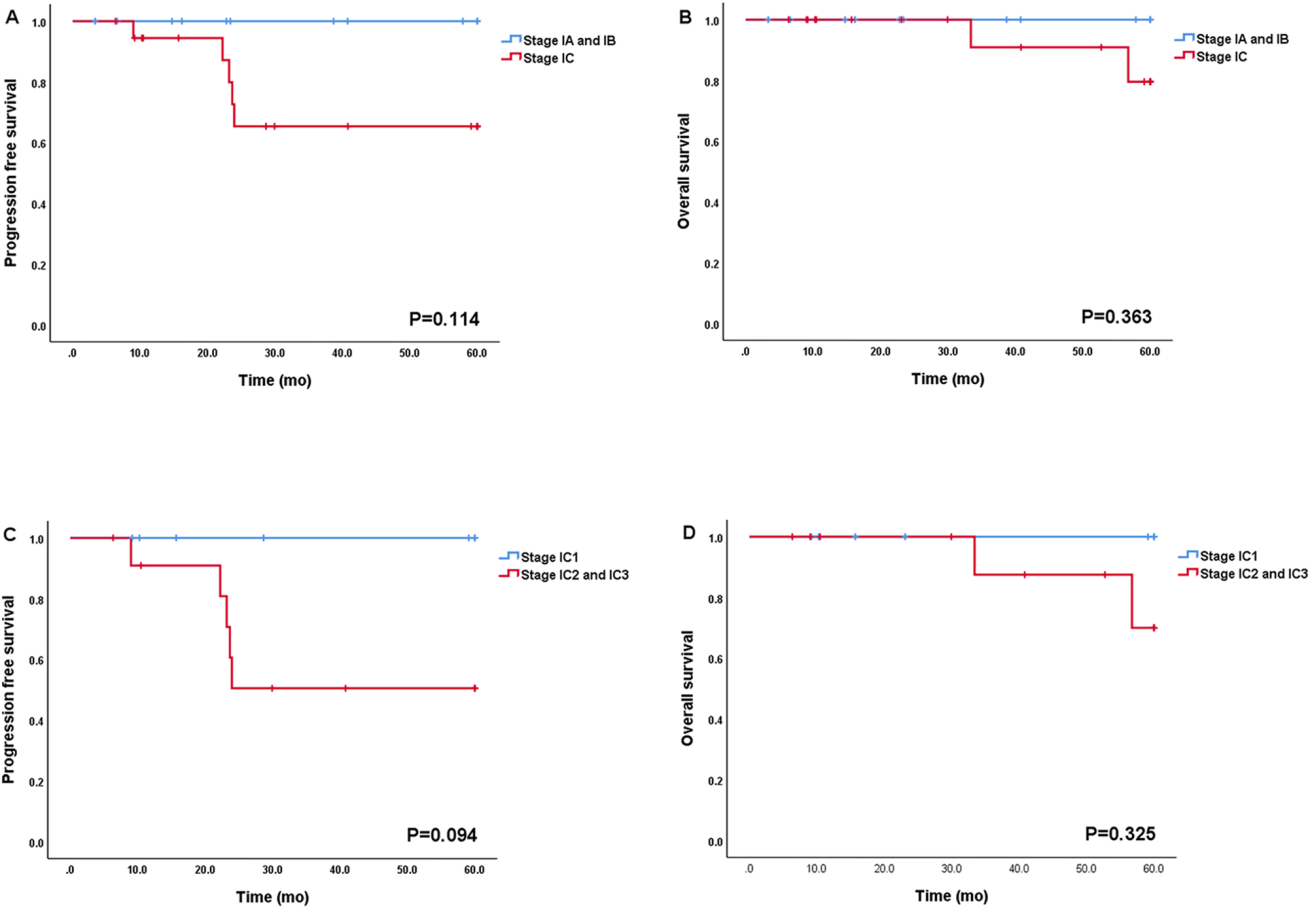
Progression-free survival and Overall survival according to FIGO stage I. (**A**) and (**B**): PFS and OS (n = 30, stage IA and IB vs. stage IC). (**C**) and (**D**): PFS and OS (n = 19, stage IC1 vs. stage IC2 and IC3).

**Figure 4:**
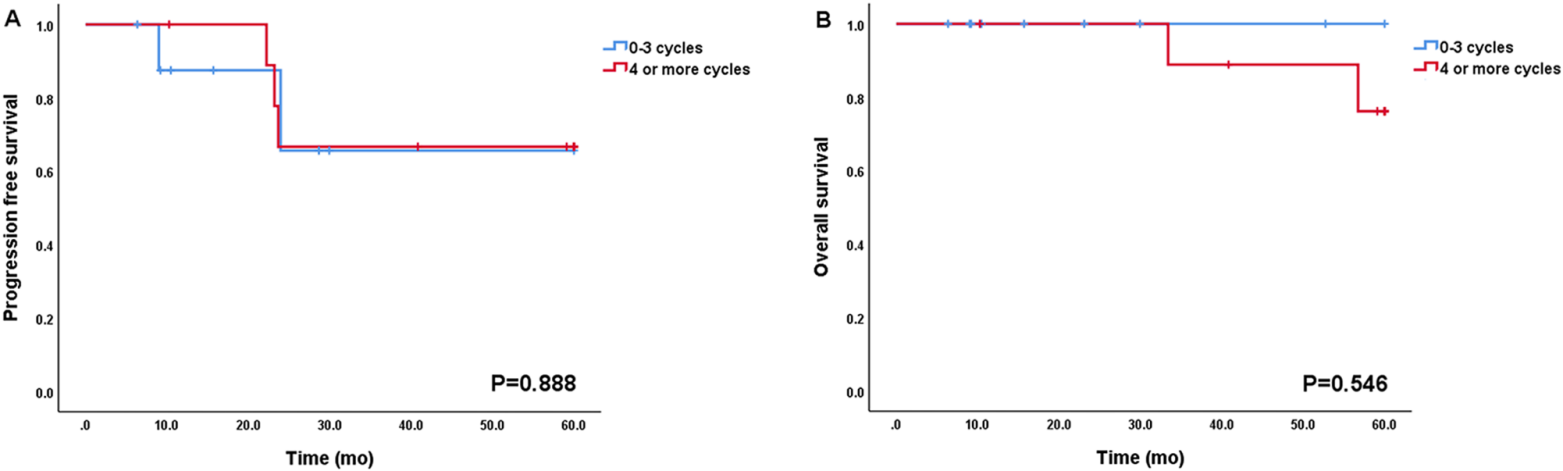
Progression-free survival and overall survival according to the number of chemotherapy cycles in FIGO stage IC (n = 19, 0–3 cycles vs. 4 or more cycles).

In subgroup analysis for FIGO stage I (n = 30), FIGO stage IA, stage IB, and stage IC were 10, 1, and 19 patients, respectively (Table 2). Twenty-one patients had complete staging surgery, 6 had limited staging mainly due to fertility preservation, and 3 did not have staging (Supplementary Table S2). The patients with FIGO stage IC experienced five recurrences (5/19, 26.3%) and two deaths (2/19, 10.5%) during follow-up period. In contrast, none of the patients experienced recurrence or death in FIGO stage IA and IB. However, such difference in prognosis did not reach statistical significance (PFS and OS, *p* = 0.114 and *p* = 0.363, respectively; Fig. 3A,B). The majority of patients with FIGO stage IA and IB (10 of 11, 91%) did not have adjuvant chemotherapy except one patient who had limited staging followed by adjuvant chemotherapy. On the other hand, most patients with FIGO stage IC received adjuvant chemotherapy except one who refused to receive adjuvant chemotherapy. Of these, 10 patients received adjuvant chemotherapy for more than three cycles (stage IC1 and IC2 vs. stage IC3, 46.1% (6/13) vs. 66.6% (4/6), *p* = 0.146). Within FIGO stage IC, all cases of recurrence (n = 5) and death (n = 2) were occurred in stage IC2 or IC3 and there was no recurrence and death in stage IC1 (PFS and OS, *p* = 0.094 and *p* = 0.325; Fig. 3C,D). However, the number of cycles of adjuvant chemotherapy for early stage disease did not affect survival (Fig. 4).

**Table 2:** The number of cycles of chemotherapy in patients with FIGO stage I.

	Stage IA (n = 10)	Stage IB (n = 1)	Stage IC1 (n = 7)	Stage IC2 (n = 6)	Stage IC3 (n = 6)
**Number of cycles of CTx**					
None	9	1	0	0	1
1–3 cycles	0	0	4	3	1
4 or more cycles	1	0	3	3	4

*CTx*. chemotherapy.

**Figure 5:**
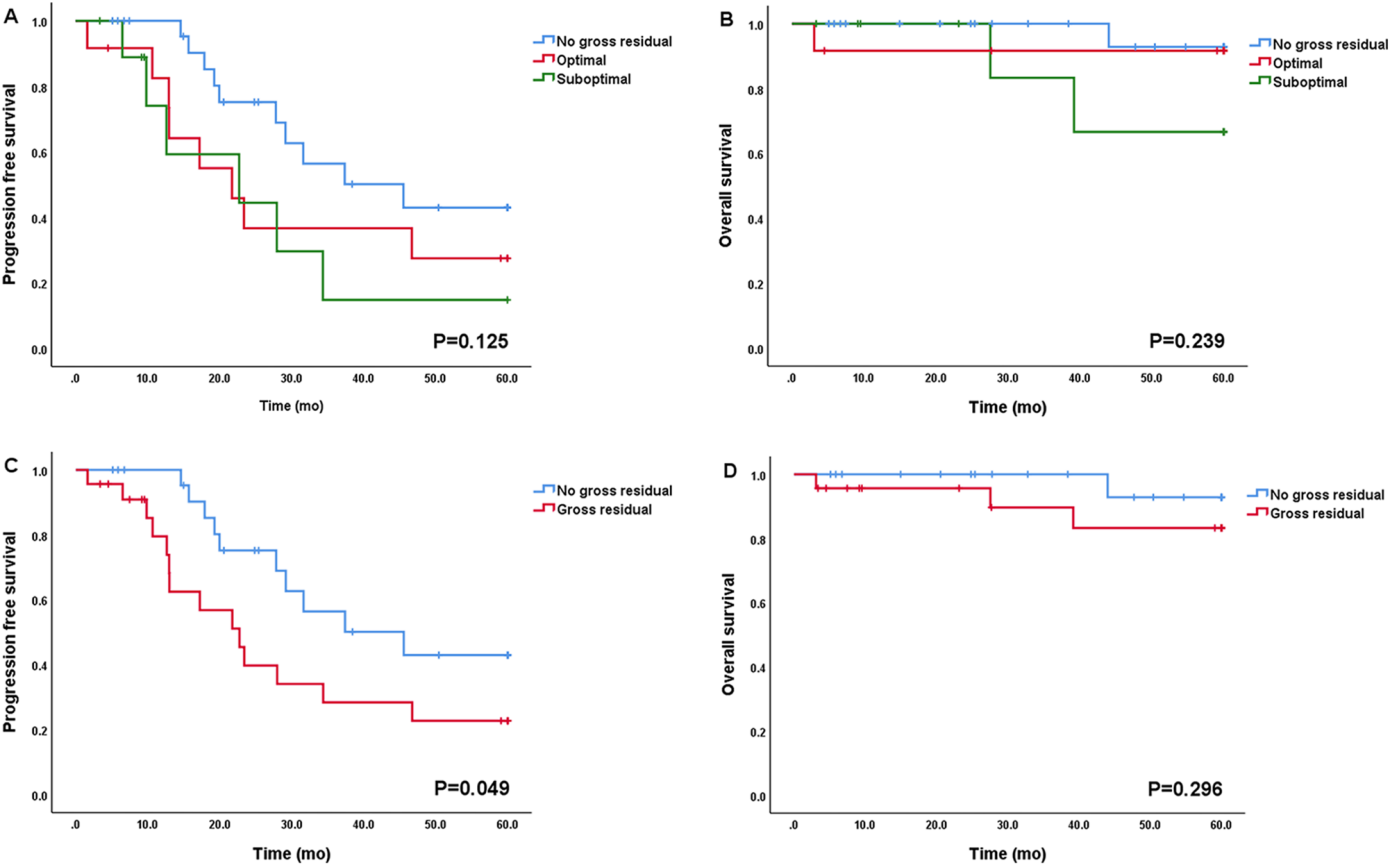
Progression-free survival and overall survival according to residual disease in FIGO stage III and IV (n = 47). (A) and (B): No gross residual vs. optimal vs. suboptimal. (C) and (D): No gross residual vs. Gross residual.

**Figure 6:**
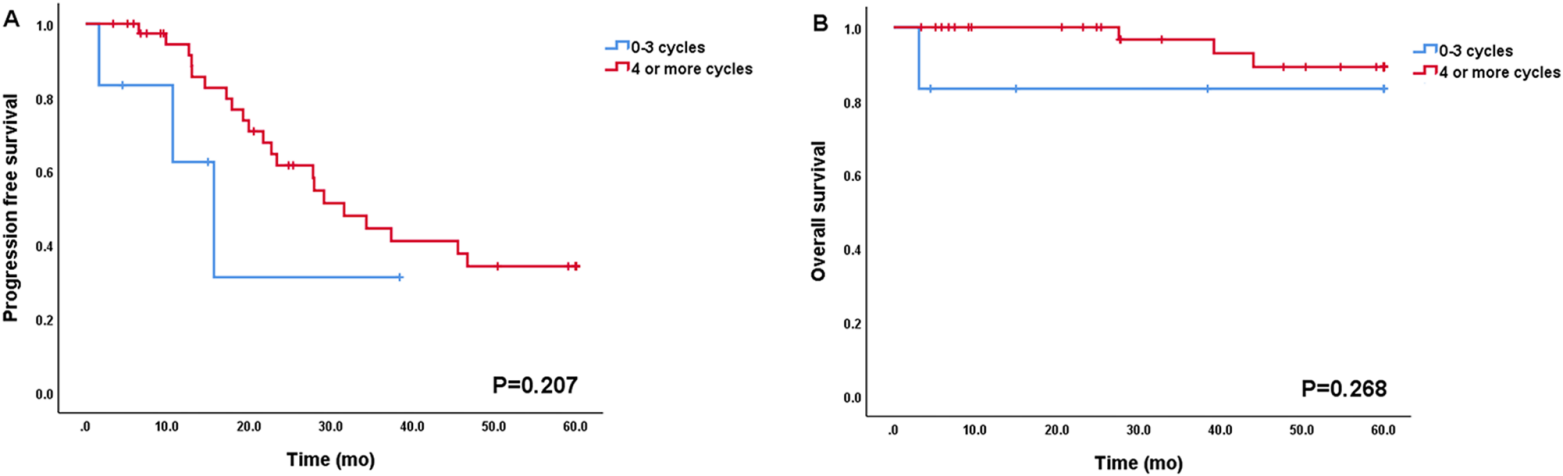
Progression-free survival and overall survival according to the number of chemotherapy cycles in FIGO stage III and IV (n = 47, 0–3 cycles vs. 4 or more cycles).

Among patients with FIGO stage III and IV (n = 47), 25 patients (53.2%) achieved no gross residual disease and 22 patients (22/47, 46.8%; 12/47, 25.5% for optimal, 10/47, 21.3% for suboptimal) had gross residual disease after primary cytoreductive surgery. As shown in Fig. 5, there was a trend wherein no gross residual disease after surgery was associated with longer PFS and OS (PFS [5A] and OS [5B], *p* = 0.125 and *p* = 0.239, respectively) when compared with those in optimal and suboptimal disease, and this trend reached statistical significance compared to no gross residual disease vs. gross residual disease for PFS (median PFS [5C]; no gross residual vs. gross residual; 26.6 months vs. 13.0 months; *p* = 0.049). Most patients with stage III and IV (45 of 47, 95.7%) received adjuvant chemotherapy, and only two patients did not because of their poor general condition after surgery. The median time interval between surgery and chemotherapy (TTC) in advanced disease was 12 days, and earlier initiation of adjuvant chemotherapy (TTC < 12 days vs. ≥ 12 days) did not affect prognosis (PFS and OS, *p* = 0.402 and *p* = 0.619). Patients who had more than three cycles of chemotherapy (n = 41) experienced 21 recurrences (21/41, 51.2%) and three deaths (3/41, 0.07%) and, in contrast to this, those who had three or less cycles of chemotherapy (n = 6) experienced three recurrences (3/6, 50%) and one death (1/6, 16.6%). However, there was no statistically significant difference (PFS and OS, *p* = 0.207 and *p* = 0.268, respectively; Fig. 6).

In a multivariate analysis (Table 3), complete resection (no gross residual) was the only significant independent prognostic factor for PFS (no gross residual vs. gross residual, HR 2.45, 95% CI 0.99–6.05, *p* = 0.045). However, it failed to reach statistical significance for OS (HR 3.60, *p* = 0.225). The number of cycles of adjuvant chemotherapy, age, preoperative CA-125 levels, and time interval of adjuvant chemotherapy were not associated with survival in patients with advanced LGSOC.

**Table 3:** Multivariate analysis of prognostic factor for PFS and OS in FIGO stage III and IV (n = 47).

Characteristics	Multivariate analysis of PFS		Multivariate analysis of OS	
	HR (95% CI)	*p*-value	HR (95% CI)	*p*-value
**Age**				
< 50	1		1	
≥ 50	1.09 (0.47–2.52)	0.842	1.00 (0.12–8.51)	1.000
**CA-125**				
< 880	1		1	
≥ 880	0.82 (0.32–2.11)	0.686	0.64 (0.12–3.47)	0.630
**TTC**				
TTC < 12	1		1	
TTC ≥ 12	0.72 (0.29–1.78)	0.470	0.84 (0.15–4.78)	0.846
**Number of cycles of CTx**				
0 ~ 3 cycle	1		1	
> 3 cycle	0.49 (0.10–2.36)	0.375	0.13 (0.01–1.18)	0.070
**Residual disease**				
No gross residual	1		1	
Gross residual	2.45 (0.99–6.05)	0.045	3.60 (0.45–28.45)	0.225

*CTx*. chemotherapy.

## Discussion

This study evaluated whether known prognostic factors in HGSOC could affect LGSOC prognosis. We observed that FIGO stage and status of residual disease after primary cytoreductive surgery for advanced disease were the independent prognostic factors for survival in LGSOC.

LGSOC has epidemiological, clinical, and molecular features distinct from those of HGSOC. First, it is a relatively rare cancer accounting for only small portion, approximately 5–10%, of the EOC and the average age at diagnosis is generally younger (55.5 years vs. 62.6 years)^15,16^. The incidence rate of LGSOC has also been decreasing steadily because of the change in the diagnostic criteria for LGSOC and borderline ovarian tumors^17^. Thus, it has not been well studied due to its rarity. Second, LGSOC shows a slowing growing pattern because of its longer cell cycle, which is a predictor of good prognosis. On the other hand, a longer cell cycle causes insensitivity to chemotherapy which is a predictor of poor prognosis^18–23^. Therefore, the role of primary debulking surgery without residual disease is emphasized more in LGSOC than in HGSOC and the finding that no gross residual disease was the only independent predictor for survival in our study is supporting this. Third, typical gene mutations of high-grade such as p53 and BRCA 1/2 are not usually detected in low-grade (8% and 5.7%)^24,25^. Another distinctive feature is the higher activation (80%) of the MAPK pathway regulated by BRAF and KRAS^26^. Furthermore, it has higher ER/PR-positive rates, which can be the theoretical background of hormonal therapy after primary treatment^19^. Based on this clinical and genetic evidence; it is considered that HGSOC and LGSOC are distinct disease entities.

In survival analysis for patients with early stage, our study (stage I and II had not reached median PFS and OS) showed relatively better survival outcomes than previous studies (median PFS and OS; 66.9 and 104.7 months^27^; 42 and 62 months^28^), even though there were only few studies for survival analysis of LGSOC patients with early stage due to its rarity. Adjuvant chemotherapy is highly recommended not only in advanced stage but also in early stage with high risk for recurrence (e.g., FIGO stage IA–IB with grade 3 or stage IC–II with any grades) in EOC with serous histology including low and high-grade. The results of two randomized controlled trials for early stage ovarian cancer conducted by the Gynecologic Oncologic Group (GOG) revealed that adjuvant chemotherapy provides survival benefit in patients with high risk. In contrast, patients with low risk (e.g., FIGO stage IA and IB, grade 1 or 2) did not have benefit from the adjuvant chemotherapy^29,30^. However, the optimal duration of chemotherapy in this disease group remains a debatable issue. The NCCN guidelines recommend three to six cycles of adjuvant chemotherapy for early disease. However, six cycles are recommended for HGSOC. A randomized GOG trial comparing three versus six cycles of adjuvant chemotherapy showed no survival benefit for extended chemotherapy despite this strategy being accompanied by increased toxicity^31^. The study conducted by Dinkelspiel et al. also reported that extended chemotherapy does not affect survival in the early stage with high risk (HR 0.93, 95% CI 0.67–1.27)^32^. Among 19 patients with FIGO stage IC in our study cohort, 18 had adjuvant chemotherapy and only one did not receive adjuvant treatment. The one patient without adjuvant chemotherapy experienced recurrence 9 months after surgery. In the extended cycle group (four or more chemotherapy cycles, n = 10), the patients had a median of six cycles of chemotherapy (range 4–6 cycles). There were three cases of recurrence, and the recurrence-free survival durations were 22.2, 23.2, and 23.6 months. In the non-extended cycle group (one to three cycles of chemotherapy, n = 8), all patients underwent three cycles of adjuvant chemotherapy and one patient experienced disease recurrence in 23.9 months after the initial treatment. Such trend in low-grade disease also consistent with the results of previous studies that extended adjuvant chemotherapy may not be required in patients with FIGO stage IC. In addition, an in vitro study revealed that LGSOC is relatively resistant to chemotherapeutic agents used to treat HGSOC^33^. These findings support that LGSOC is a slow growing tumor and relatively chemoresistant as opposed to HGSOC. As a result, we could carefully speculate that extended chemotherapy or any chemotherapy may not be beneficial at all in patients with low-grade disease. However, there is still lack of evidence supporting these. Currently, the clinical trial (NRG-GY019)^34^ comparing the survival outcomes between adjuvant chemotherapy and adjuvant hormone therapy in LGSOC is recruiting patients. Although early stage is excluded in this study, it is highly expected that treatment specialized for LGSOC will be established depending on the results of this study, even though this trial cannot answer the question about the role of adjuvant chemotherapy against no treatment. Further study is needed to determine the benefit of chemotherapy in LGSOC.

It is well known that the amount of residual disease after the primary debulking surgery is a very strong prognostic factor for survival in EOC^35–37^. According to the analysis of 5114 patients at advanced stage EOC conducted by the AGO (Arbeitsgemeinschaft Gynaekologische Onkologie) group^38^, complete cytoreduction (no gross residual) was achieved in 52% and 35% of patients with LGSOC and HGSOC, respectively. When comparing survival between patients with low-grade and high-grade disease according to optimality, for LGSOC, survival benefit was observed only in the group with no gross residual (low-grade vs. high-grade; 5-year OS: 85% vs. 61%; *p* = 0.001). There was no survival benefit in the gross residual group when compared with that in HGSOC^38^, which demonstrated the importance of complete gross resection in LGSOC. According to their study, 5-year OS was 85% for the LGSOC patients who underwent complete cytoreduction versus 32% for those with suboptimal. This finding corresponds well with that of our study (5-year OS; 91% and 65% for the patients who achieve no gross residual and suboptimal, respectively; Fig. 5). In a multivariate analysis, we found no gross residual disease was the only significant independent prognostic factor for PFS (gross residual vs. no gross residual, HR: 2.45 [95% CI 0.99–6.05], *p* = 0.045). Similarly, the AGO group also revealed that achieving complete cytoreduction is an independent prognostic factor for PFS and OS (HR 0.447 and HR 0.144, *p* < 0.001). Although we could not observe statistical significance in OS (Fig. 5D), partly owing to a few events, our findings suggest that complete cytoreduction is an indisputable prognostic factor in LGSOC. Considering its relatively high chemoresistance, the importance of surgery including complete surgical staging and debulking in LGSOC must be more emphasized.

Our study also revealed that the time interval between surgery and adjuvant chemotherapy was not associated with survival in the advanced stages of LGSOC (Table 3). The optimal timing of chemotherapy has been a controversial issue in HGSOC^39^. There has been a long-standing issue that surgery-induced accelerated tumor growth may lead to poor prognosis and demised survivals on solid tumors from delayed adjuvant chemotherapy after surgery supports this. As the earlier initiation of adjuvant chemotherapy may alleviate this negative effect of surgery, TTC is considered a prognostic factor in HGSOC^40–42^. For LGSOC, no study was conducted to see the effect of TTC on survival amid its biologic difference (relatively slow growing tumor of LGSOC) and different platinum sensitivity (relatively insensitive to platinum) from those of HGSOC, which may affect the effect of TTC on prognoses. We think that comparing survival outcomes according to TTC between various centers with different TTC will be a valuable study and further large sample size studies are needed in the future.

The strength of this study is that various clinical factors were analyzed only for low-grade disease and that the study patients were included from two cancer centers in different countries. Similar to previous studies (Supplementary Table S3), our study reaffirmed the prognostic significance of FIGO stage and residual disease status in low-grade disease. However, in particular, we analyzed the role of chemotherapy for early stage and the prognostic value of TTC for advanced stage, which have not been studied yet in low-grade disease. However, there are some limitations. First, this was a retrospective study. Second, the sample size was small. Third, there was no central gynecologic pathologic review, which could have resulted in inaccurate diagnoses in some patients^43^. Since the two-tier system was announced in 2004^3,4^, we presented the analysis results for patients (n = 77) excluding those diagnosed before that time (n = 7) as supplementary materials (Supplementary Figs. S2–S6 and Supplementary Table S4). Furthermore, there was no evaluation in terms of molecular and genetic factors. Recently, the use of aromatase inhibitor has been proposed as a new treatment option for ER-positive LGSOC. Further, clinical trials are being actively conducted to evaluate the effect of letrozole in treating patients with LGSOC (NRG-GY019)^34^. However, there was no information and analysis of hormone therapy in this study, which is another weakness of our study.

In conclusion, our study revealed that the known prognostic factors in HGSOC did not show any significant effect on survival in low-grade disease except for the FIGO stage and residual disease status. Further studies are essential to understand the clinical behavior of this unique disease in EOC.

## Supplementary information

Supplementary Tables.

Supplementary Dataset.

Supplementary Figure 1.

Supplementary Figure 2.

Supplementary Figure 3.

Supplementary Figure 4.

Supplementary Figure 5.

Supplementary Figure 6.

Supplementary Legends.

## Data Availability

The datasets analyzed during the current study are available from the corresponding author on reasonable request.
