# Supplementary material for: Clinical factors associated with prognosis in low-grade serous ovarian carcinoma: experiences at two large academic institutions in Korea and Taiwan
Source: Sci Rep. 2020 Nov 17;10:20012. doi: 10.1038/s41598-020-77075-1 (PMC7672053; doi:10.1038/s41598-020-77075-1)
Supplement: Supplementary file 1 — Supplementary Tables. [file 41598_2020_77075_MOESM1_ESM.docx]

**Clinical factors associated with prognosis in low-grade serous ovarian carcinoma: Experiences at two** **large academic institutions in Korea and Taiwan**

Jun-Hyeok Kang, Yen-Ling Lai, Wen-Fang Cheng, Hyun-Soo Kim, Kuan-Ting Kuo, Yu-Li Chen, Yoo-Young Lee

**Supplementary Table S1.** Patients and treatment characteristics according to institution.

| **SMC** | Total  (n = 64) | Stage I  (n = 27) | Stage II  (n = 5) | Stage III  (n = 30) | Stage IV  (n = 2) | *p*-value |
| --- | --- | --- | --- | --- | --- | --- |
| Age(years) |  |  |  |  |  |  |
| Mean ± SD | 47.7 ± 14.7 | 45.0 ± 16.8 | 48.4 ± 13.2 | 49.8 ± 13.3 | 50.0 ± 4.2 | 0.661 |
| CA-125(U/mL) |  |  |  |  |  |  |
| Median(IQR) | 137.1 (27.7-648.5) | 49.8 (11.3-121.8) | 128.8 (40-352.9) | 474.2 (134.0-1918.4) | 3345 (2100.0-4590.3) | 0.048^*^ |
| Residual disease |  |  |  |  |  | 0.044^*^ |
| No gross residual | 56(87.5%) | 27(100%) | 5(100%) | 23(76.7%) | 1(50%) |  |
| Optimal (< 1cm) | 5(7.8%) | 0 | 0 | 4(13.3%) | 1(50%) |  |
| Suboptimal (≥1cm) | 3(4.7%) | 0 | 0 | 3(10.0%) | 0 |  |
| TTC(days) |  |  |  |  |  |  |
| Median(IQR) | 11(9-18) | 12(9-18) | 10(9-12) | 10(9-15) | 31 | 0.081 |
| Number of cycles of CTx. |  |  |  |  |  | 0.002^*^ |
| None | 12(18.8%) | 10(37.0%) | 0 | 2(6.7%) | 0 |  |
| 1-3 cycles | 10(15.6%) | 7(26.0%) | 0 | 2(6.7%) | 1(50.0%) |  |
| 4 or more cycles | 42(65.6%) | 10(37.0%) | 5(100%) | 26(86.6%) | 1(50.0%) |  |
| **NTUH** | Total  (n = 20) | Stage I  (n = 3) | Stage II  (n = 2) | Stage III  (n = 14) | Stage IV  (n = 1) | *p*-value |
| Age(years) |  |  |  |  |  |  |
| Mean ± SD | 53.4 ± 17.6 | 60.7 ± 20.2 | 34.5 ± 20.5 | 52.4 ± 14.7 | 84.0 | 0.106 |
| CA-125(U/mL) |  |  |  |  |  |  |
| Median(range) | 890.1 (329.6-2435.3) | 347.1 (193.5-1881.5) | 262.2 (254.7-269.7) | 1055 (518.4-3192.2) | 2108.4 | 0.153 |
| Residual disease |  |  |  |  |  | 0.009^*^ |
| No gross residual | 6(30.0%) | 3(100.0%) | 2(100.0%) | 1(7.1%) | 0 |  |
| Optimal (< 1cm) | 7(35.0%) | 0 | 0 | 7(50.0%) | 0 |  |
| Suboptimal (≥1cm) | 7(35.0%) | 0 | 0 | 6(42.9%) | 1(100.0%) |  |
| TTC(days) |  |  |  |  |  |  |
| Median(IQR) | 14(11-20) | 9(8-10) | 19 | 14(11-19) | 21 | 0.231 |
| Number of cycles of CTx. |  |  |  |  |  | 0.006^*^ |
| None | 2(10%) | 1(33.3%) | 1(50.0%) | 0 | 0 |  |
| 1-3 cycles | 2(10%) | 1(33.3%) | 0 | 1(7.1%) | 0 |  |
| 4 or more cycles | 16(80%) | 1(33.3%) | 1(50.0%) | 13(92.9%) | 1(100.0%) |  |

SMC, Samsung Medical Center; NTUH, National Taiwan University Hospital; TTC, time from the surgery to adjuvant chemotherapy; IQR, interquartile range; CTx., chemotherapy.

^*^*p*-value is less than 0.05.

**Supplementary Table S2.** Brief medical history of the patients with FIGO stage I.

| **No.** | **Year** | **Age** | **Stage** | **CA-125**  **(U/mL)** | **Op. name** | **Adjuvant treatment** | **Status of surgical staging** | **Post-operative imaging** |
| --- | --- | --- | --- | --- | --- | --- | --- | --- |
| 1 | 2005 | 44 | IA | 50 | RSO (incidental) | 4 cycles chemotherapy  (without re-staging op.) | Unstaged | NED |
| 2 | 2008 | 31 | IC2 | 23 | LAVH, BSO (incidental) | 6 cycles chemotherapy  (without re-staging op.) | Unstaged | NED |
| 3 | 2008 | 56 | IC3 | 644 | TAH, BSO, PLND, OM | 6 cycles chemotherapy | Complete |  |
| 4 | 2009 | 50 | IC2 | 160 | LAVH, BSO, PLND, PALND, OM, Washing cytology | 6 cycles chemotherapy | Complete |  |
| 5 | 2009 | 26 | IC1 | 252 | P/LSO, Right ovary biopsy,  PLND, OM (fertility sparing) | 4 cycles chemotherapy | Limited | NED |
| 6 | 2013 | 28 | IC3 | 8.6 | P/LSO (incidental) → P/ROC, PLND, PALND, OM, Washing cytology (re-staging / fertility sparing) | 6 cycles chemotherapy | Limited | NED |
| 7 | 2015 | 13 | IC2 | 330 | P/LSO, OM, washing cytology  (fertility sparing) | 3 cycles chemotherapy | Limited | NED |
| 8 | 2015 | 40 | IC3 | 224 | TAH, BSO, OM, Washing cytology | 4 cycles chemotherapy | Complete |  |
| 9 | 2012 | 41 | IC1 | 12 | LAVH,BSO (incidental) | 6 cycles chemotherapy  (without re-staging op.) | Unstaged | NED |
| 10 | 2001 | 19 | IC3 | 74 | TAH, BSO, OM, Washing cytology | 6 cycles chemotherapy | Complete |  |
| 11 | 2015 | 25 | IC2 | 630 | TAH, BSO, OM, Washing cytology | 3 cycles chemotherapy | Complete |  |
| 12 | 2018 | 44 | IC1 | 4.1 | LAVH, BSO, PLND, OM, Washing cytology | 3 cycles chemotherapy | Complete |  |
| 13 | 2016 | 52 | IC1 | 4.5 | TLH, BSO, PLND, OM, Washing cytology | 3 cycles chemotherapy | Complete |  |
| 14 | 2018 | 62 | IC1 | 20 | TAH, BSO, PLND, OM, Washing | 4 cycles chemotherapy | Complete |  |
| 15 | 2018 | 58 | IC2 | 24 | TAH, BSO, PLND, OM, Washing | 3 cycles chemotherapy | Complete |  |
| 16 | 2018 | 60 | IC1 | 4 | TLH, BSO, PLND, OM, Washing cytology | 3 cycles chemotherapy | Complete |  |
| 17 | 2018 | 32 | IC3 | 50 | P/LSO, OM, Washing cytology  (fertility sparing) | 3 cycles chemotherapy | Limited | NED |
| 18 | 2010 | 50 | IC1 | 40 | TAH, BSO, OM, Washing cytology | 3 cycles chemotherapy | Complete |  |
| 19 | 2010 | 48 | IC2 | 341 | TAH, BSO, OM, PLND, Washing cytology | 6 cycles chemotherapy | Complete |  |
| 20 | 2015 | 52 | IA | 144 | TAH, BSO, OM, Washing cytology | N/A | Complete |  |
| 21 | 2014 | 60 | IB | 10 | TAH, BSO, OM, Washing cytology | N/A | Complete |  |
| 22 | 2017 | 62 | IA | 128 | TAH, BSO, OM, Washing cytology | N/A | Complete |  |
| 23 | 2013 | 21 | IA | 18 | P/LSO, OM, Washing cytology  (fertility sparing) | N/A | Limited | NED |
| 24 | 2012 | 57 | IA | 8.6 | TAH, BSO, PLND, OM, Washing cytology | N/A | Complete |  |
| 25 | 2017 | 85 | IA | 60 | TAH, BSO, OM, Washing cytology | N/A | Complete |  |
| 26 | 2011 | 52 | IA | 5.1 | P/LSO (incidental) → TAH, RSO, OM, Washing cytology (re-staging) | N/A | Complete |  |
| 27 | 2016 | 57 | IA | 92 | TAH, BSO, PLND, OM, Washing cytology | N/A | Complete |  |
| 28 | 2017 | 33 | IA | 74 | P/RSO (incidental) →  P/PLND, OM, Washing cytology (re-staging / fertility sparing) | N/A | Limited | NED |
| 29 | 2014 | 54 | IC3 | 12.1 | TAH, BSO, OM, PLND, Washing cytology | Refusal of adjuvant treatment | Complete |  |
| 30 | 2015 | 84 | IA | 347 | TAH, BSO, OM, Washing cytology | N/A | Complete |  |

Op., operation; RSO, right salpingo-oophorectomy; NED; no evidence of disease; LAVH, laparoscopic assisted vaginal hysterectomy; BSO, both salpingo-oophorectomy; TAH; total abdominal hysterectomy; PLND, pelvic lymph node dissection; OM, omentectomy; PALND, para-aoritc lymph node dissection; P, pelviscopy; LSO, left salpingo-oophorectomy; ROC, right ovarian cystectomy; TLH; total laparoscopic hysterectomy; N/A, not applicable.

**Supplementary Table S3.** Literature review of studies that evaluate prognostic factor for LGSOC.

| **Author** | **Year** | **Design** | **No.** | **FIGO stage** | **Outcome measurement** | **Treatment** | **Prognostic factor** | **Survival outcome (P < 0.05)** | | |
| --- | --- | --- | --- | --- | --- | --- | --- | --- | --- | --- |
|  |  |  |  |  |  |  |  | **Variable** | **PFS (month)** | **OS (month)** |
| Grabowski et al. [1] | 2016 | Retrospective | 145 | IIIB-IV | Survival | CRS  CTx. | RD after CRS | RD > 1cm | Data not shown | 35.0 |
|  |  |  |  |  |  |  |  | RD ≤ 1cm | Data not shown | Data not shown |
|  |  |  |  |  |  |  |  | RD = 0 | Data not shown | 97.0 |
| Gershenson et al. [2] | 2015 | Retrospective | 350 | I-IV | Survival | CRS  CTx. | Disease status at completion of primary treatment* | NED | 33.4 | 112.9 |
|  |  |  |  |  |  |  |  | Disease present | 17.2 | 74.6 |
|  |  |  |  |  |  |  | Age ≤ 35 years | Age ≤ 35 | 17.8 | 72.8 |
|  |  |  |  |  |  |  |  | Age > 35 | 31.2 | 102.9 |
| Chen et al.[3] | 2014 | Retrospective | 35 | I-III | Survival | CRS  CTx. | FIGO stage | I-II | 42 | 61 |
|  |  |  |  |  |  |  |  | III-IV | 11 | 44 |
|  |  |  |  |  |  |  | RD after CRS | RD ≤ 1cm | 36 | 56 |
|  |  |  |  |  |  |  |  | RD > 1cm | 10 | 30 |
| Fader et al. [4] | 2013 | Subgroup analysis of GOG 182 | 189 | III-IV | Survival | CRS  CTx. | RD after CRS | RD = 0 | 33.2 | 96.9 |
|  |  |  |  |  |  |  |  | RD < 1cm | 14.7 | 44.5 |
|  |  |  |  |  |  |  |  | RD ≥ 1cm | 14.1 | 42.0 |
| Gershenson et al. [5] | 2006 | Retrospective | 112 | II-IV | Survival | CRS  CTx. | Disease status at completion of primary treatment* | NED | 26.0 | 102.9 |
|  |  |  |  |  |  |  |  | Disease present | 14.5 | 47.0 |

No., number of patient; PFS, progression-free survival; OS, overall survival; CRS, cytoreductive surgery; HT, hormone therapy; CTx., chetmotherapy; RD, residual disease; NED, no evidence of disease; GOG, gynecologic oncology group.

1. Grabowski JP, Harter P, Heitz F et al. Operability and chemotherapy responsiveness in advanced low-grade serous ovarian cancer. An analysis of

the AGO Study Group metadatabase. Gynecol Oncol 2016; 140: 457-462.

2. Gershenson DM, Bodurka DC, Lu KH et al. Impact of Age and Primary Disease Site on Outcome in Women With Low-Grade Serous Carcinoma

of the Ovary or Peritoneum: Results of a Large Single-Institution Registry of a Rare Tumor. J Clin Oncol 2015; 33: 2675-2682.

3. Chen M, Jin Y, Bi Y et al. A survival analysis comparing women with ovarian low-grade serous carcinoma to those with high-grade histology.

OncoTargets and therapy 2014; 7: 1891-1899.

4. Fader AN, Java J, Ueda S et al. Survival in women with grade 1 serous ovarian carcinoma. Obstetrics and gynecology 2013; 122: 225-232.

5. Gershenson DM, Sun CC, Lu KH et al. Clinical behavior of stage II-IV low-grade serous carcinoma of the ovary. Obstet Gynecol 2006; 108: 361-

368.

**References**

**Supplementary Table S4.** Multivariate analysis of prognostic factor for PFS and OS in FIGO stage III and IV (n = 42).

| Characteristics | | Multivariate analysis of PFS | | Multivariate analysis of OS | |
| --- | --- | --- | --- | --- | --- |
|  |  | HR(95% CI) | *p*-value | HR(95% CI) | *p*-value |
| Age | < 50 | 1 |  | 1 |  |
|  | ≥ 50 | 1.19(0.46-3.087) | 0.713 | 1.04(0.35-8.75) | 0.975 |
| CA-125 | < 880 | 1 |  | 1 |  |
|  | ≥ 880 | 0.86(0.29-2.51) | 0.786 | 1.76(0.15-10.51) | 0.651 |
| TTC | TTC < 12 | 1 |  | 1 |  |
|  | TTC ≥ 12 | 0.82(0.31-2.15) | 0.687 | 1.53(0.17-5.43) | 0.567 |
| Number of cycles of CTx. | 0~3 cycle | 1 |  | 1 |  |
|  | > 3 cycle | 0.57(0.12-2.77) | 0.493 | 0.42(0.11-1.24) | 0.084 |
| Residual disease | No gross residual | 1 |  | 1 |  |
|  | Gross residual | 2.33(1.05-6.12) | 0.041 | 3.45(0.57-14.73) | 0.257 |

CTx., chemotherapy.
