# Supplementary figures and images for: Clinical factors associated with prognosis in low-grade serous ovarian carcinoma: experiences at two large academic institutions in Korea and Taiwan
Source: Sci Rep. 2020 Nov 17;10:20012. doi: 10.1038/s41598-020-77075-1 (PMC7672053; doi:10.1038/s41598-020-77075-1)

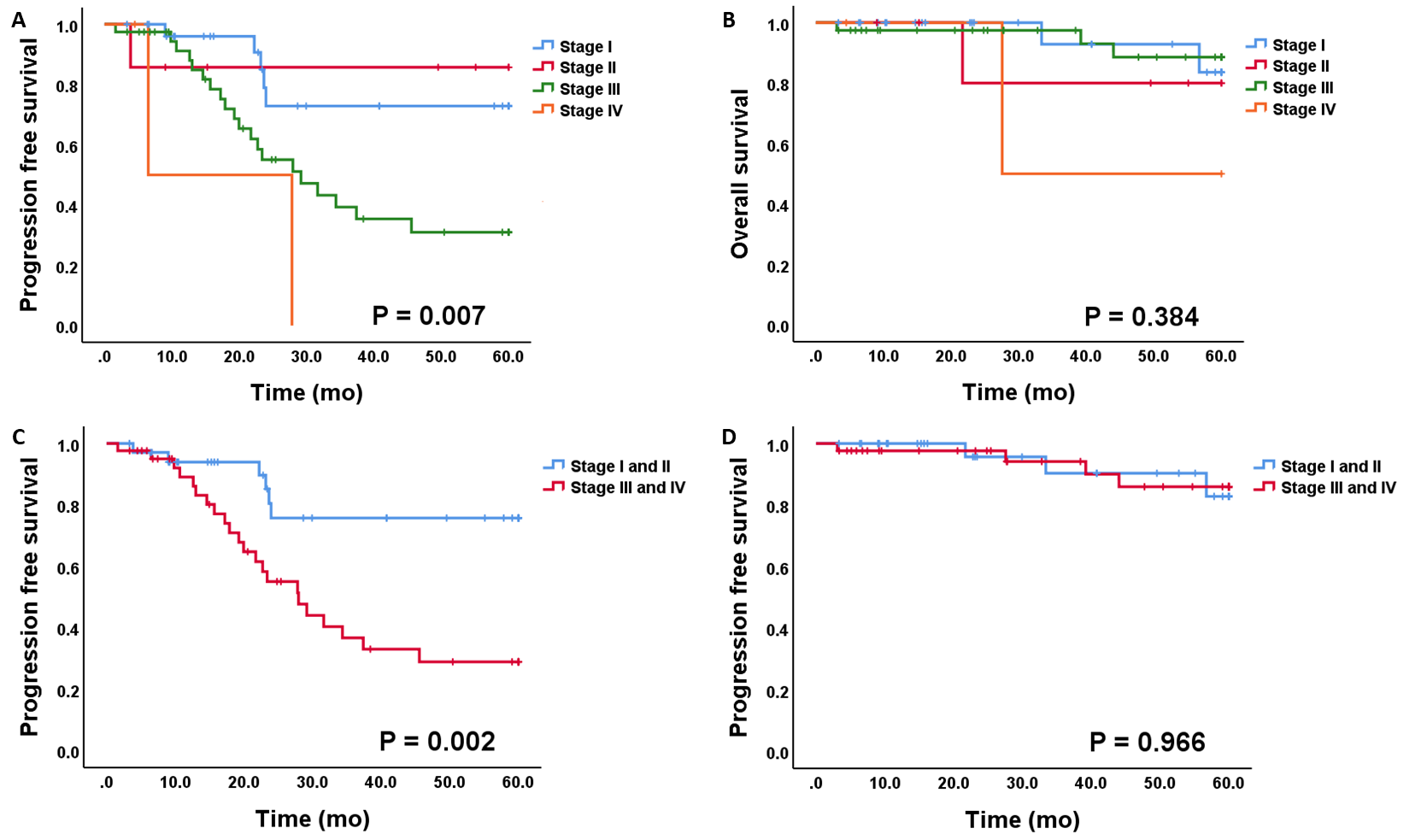

Supplement: Supplementary file 3 — Supplementary Figure 1. [file 41598_2020_77075_MOESM3_ESM.tif]

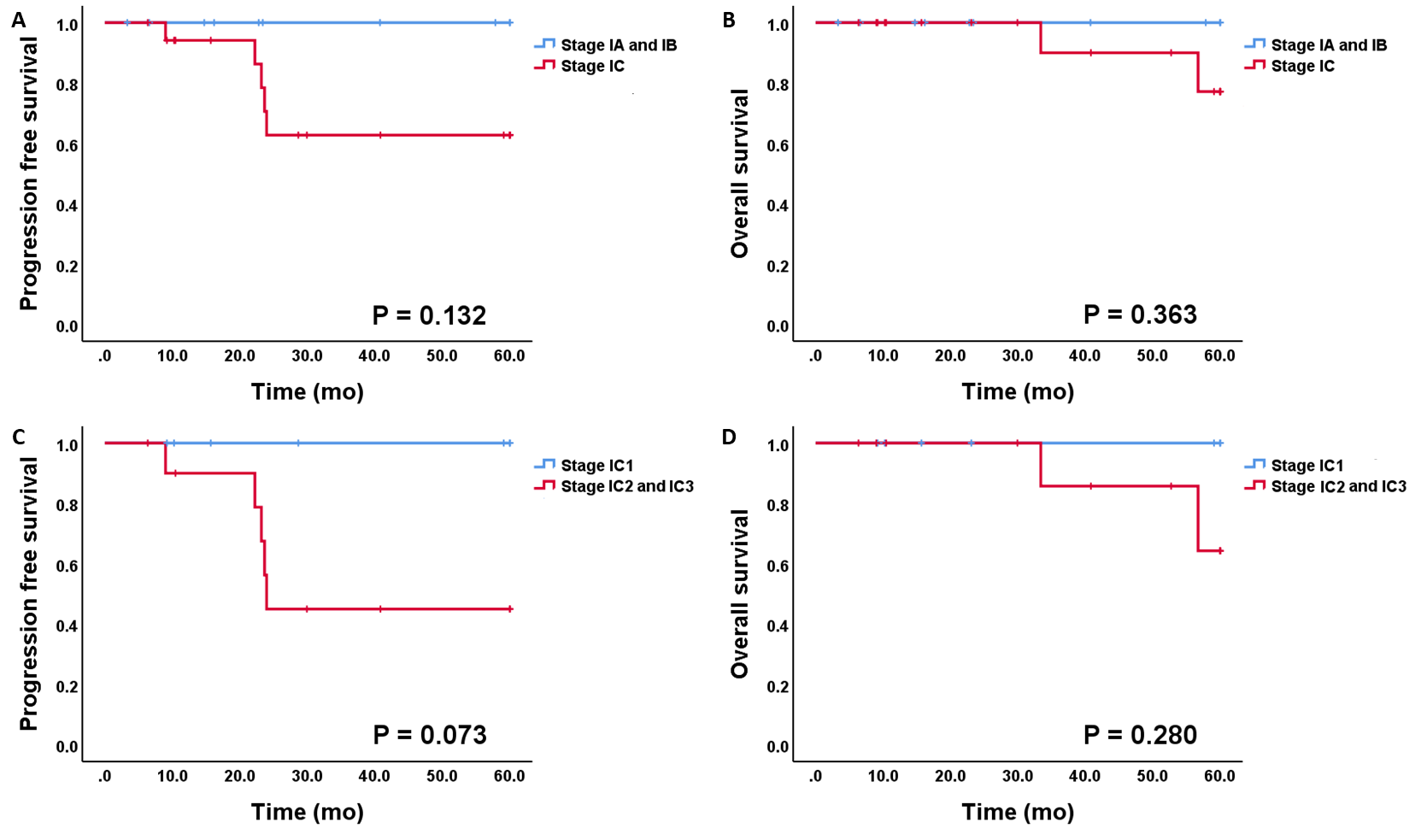

Supplement: Supplementary file 4 — Supplementary Figure 2. [file 41598_2020_77075_MOESM4_ESM.tif]

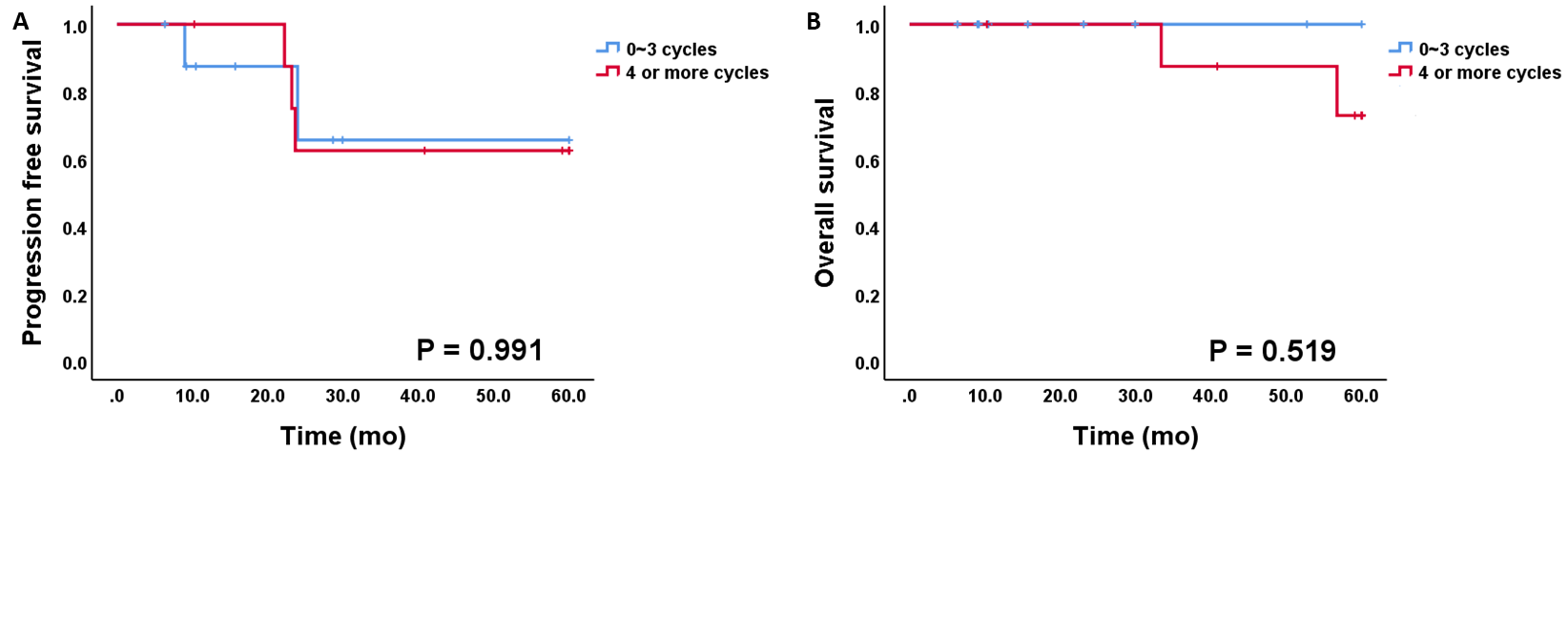

Supplement: Supplementary file 5 — Supplementary Figure 3. [file 41598_2020_77075_MOESM5_ESM.tif]

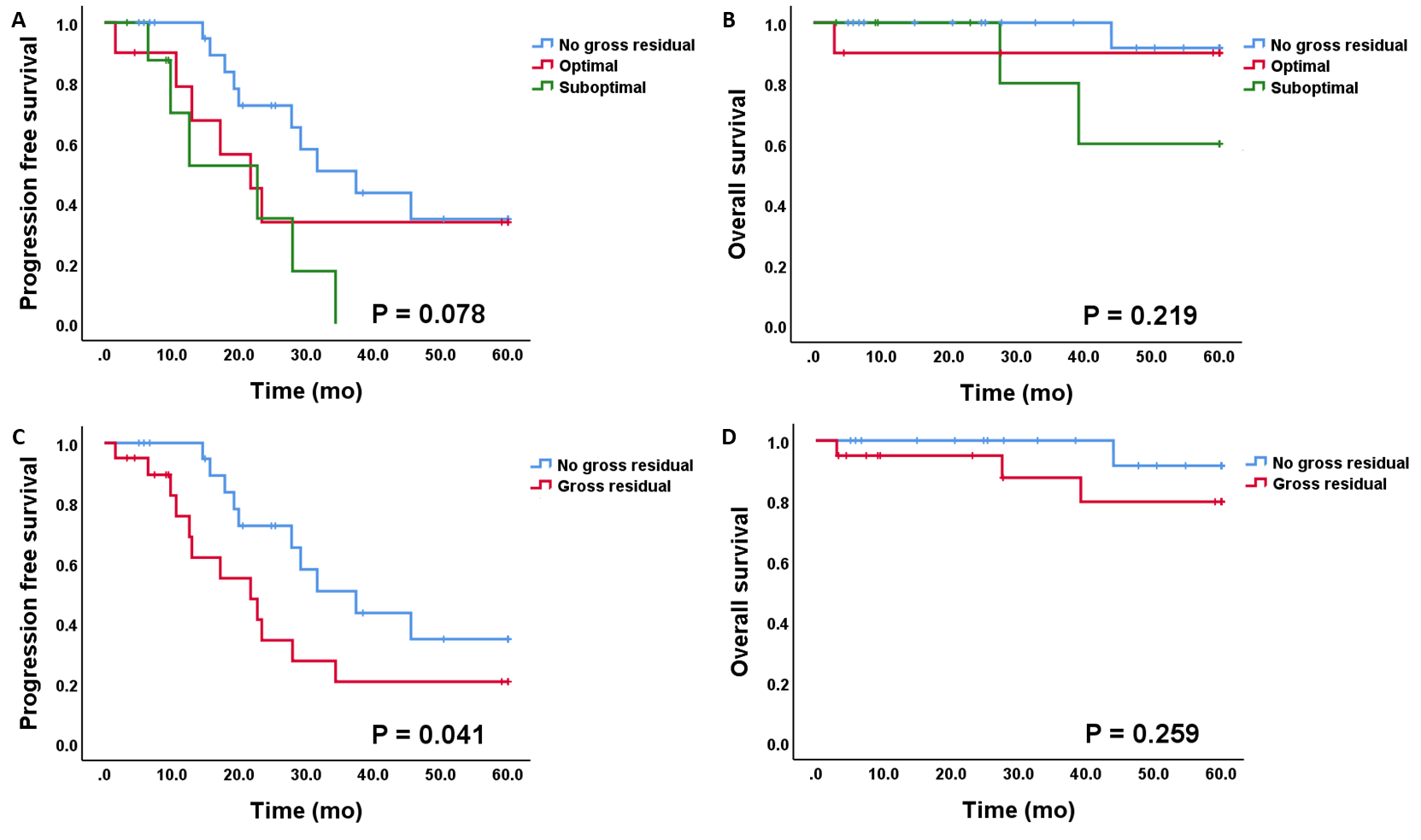

Supplement: Supplementary file 6 — Supplementary Figure 4. [file 41598_2020_77075_MOESM6_ESM.tif]

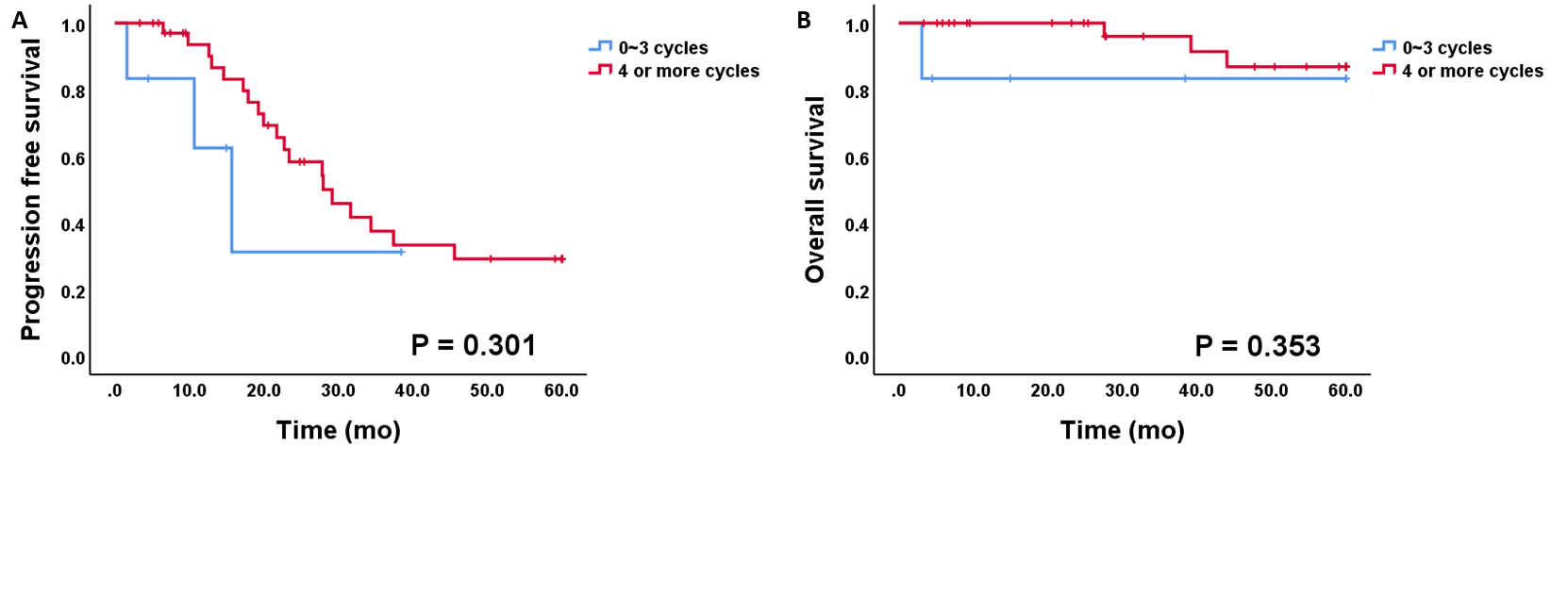

Supplement: Supplementary file 7 — Supplementary Figure 5. [file 41598_2020_77075_MOESM7_ESM.tif]

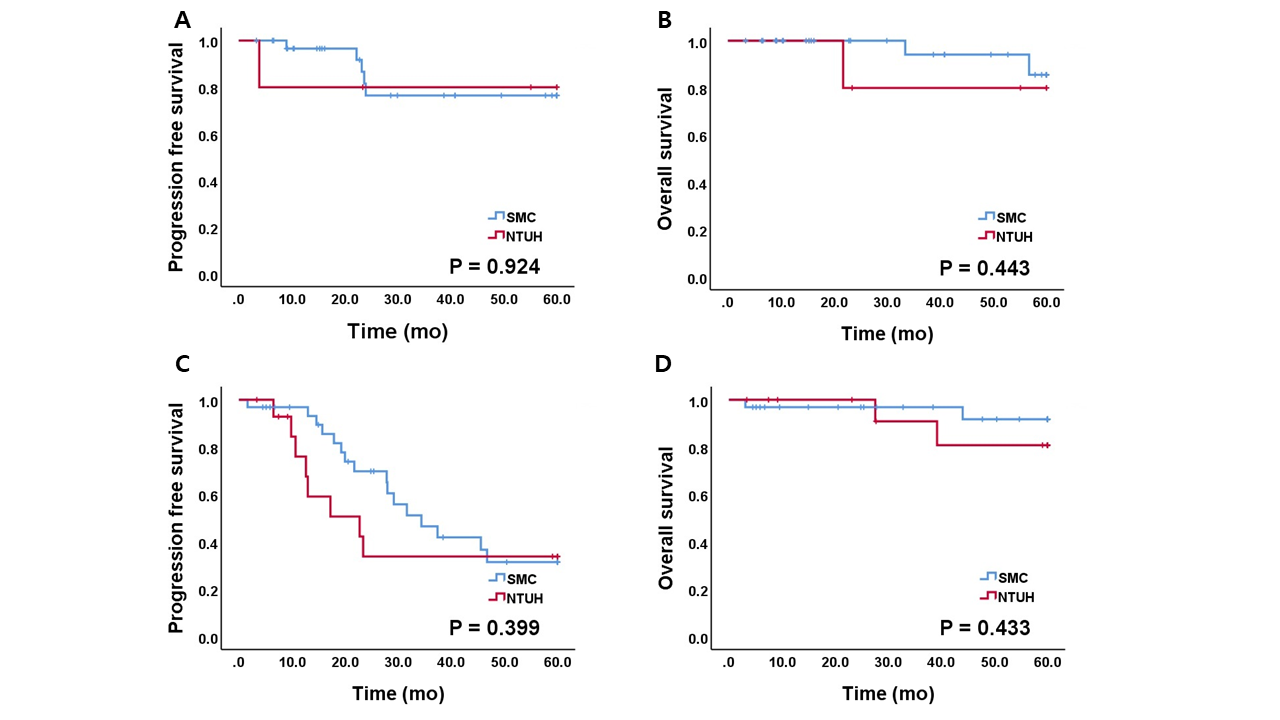

Supplement: Supplementary file 8 — Supplementary Figure 6. [file 41598_2020_77075_MOESM8_ESM.tif]
