# Supplementary material for: Clinical factors associated with prognosis in low-grade serous ovarian carcinoma: experiences at two large academic institutions in Korea and Taiwan
Source: Sci Rep. 2020 Nov 17;10:20012. doi: 10.1038/s41598-020-77075-1 (PMC7672053; doi:10.1038/s41598-020-77075-1)
Supplement: Supplementary file 9 — Supplementary Legends. [file 41598_2020_77075_MOESM9_ESM.docx]

**Supplementary Figure Legend**

**Supplementary Figure S1.** Progression-free survival and overall survival according to institution.

(A) and (B): FIGO stage I and II (SMC [n=32] vs. NTUH [n=5]). (C) and (D): FIGO stage III and IV (SMC [n=32] vs. NTUH [n=15]).

SMC, Samsung Medical Center; NTUH, National Taiwan University Hospital.

**Supplementary Figure S2.** Progression-free survival and overall survival according to FIGO stage (all patients [n=77]; stage I [n=28], stage II [n=7], stage III [n=39], and stage IV [n=3]).

**Supplementary Figure S3.** Progression-free survival and overall survival according to FIGO stage I.

(A) and (B): PFS and OS (n=28, stage IA and IB [n=10] vs. stage IC [n=18])

(C) and (D): PFS and OS (n=18, stage IC1 [n=7] vs. stage IC2 and IC3 [n=11])

**Supplementary Figure S4.** Progression-free survival and overall survival according to the number of chemotherapy cycles in FIGO stage IC (n = 18, 0-3 cycles [n=9] vs. 4 or more cycles [n=9]).

**Supplementary Figure S5.** Progression-free survival and overall survival according to residual disease in FIGO stage III and IV (n = 42).

(A) and (B): No gross residual [n=22 vs. optimal [n=11] vs. suboptimal [n=9]. (C) and (D): No gross residual [n=22] vs. Gross residual [n=20].

**Supplementary Figure S6.** Progression-free survival and overall survival according to the number of chemotherapy cycles in FIGO stage III and IV (n = 42, 0-3 cycles [n=6] vs. 4 or more cycles [n=36]).
